# Effects of P-Glycoprotein and Its Inhibitors on Apoptosis in K562 Cells

**DOI:** 10.3390/molecules190913061

**Published:** 2014-08-25

**Authors:** Yaqiong Zu, Zhiyong Yang, Songshan Tang, Ying Han, Jun Ma

**Affiliations:** 1 Department of Health Statistics, School of Public Health, Tianjin Medical University, 22 Qixiangtai Road, Heping District, Tianjin 300070, China; E-Mail: zuyq@tijmu.edu.cn; 2 HUYA Bioscience International LLC, 3 Haidian Avenue, Haidian District, Beijing 100080, China; E-Mail: yzy_lz@hotmail.com; 3 Department of Biochemistry and Molecular Biology, School of Basic Courses, Guangdong Pharmaceutical University, 280 Waihuandong Road, Guangzhou Higher Education Mega Center, Guangzhou 510006, China; E-Mail: songstang@hotmail.com; 4 Biotherapy Center of Tianjin Cancer Institute and Hospital, Tianjin Medical University, Tiyuanbei, Huanhuxi Road, Hexi District, Tianjin 300060, China; E-Mail: 13612190539@163.com

**Keywords:** P-glycoprotein, apoptosis, K562 cells, P-glycoprotein inhibitors, caspase 3, PSC833, verapamil, H108

## Abstract

P-glycoprotein (P-gp) is a major factor in multidrug resistance (MDR) which is a serious obstacle in chemotherapy. P-gp has also been implicated in causing apoptosis of tumor cells, which was shown to be another important mechanism of MDR recently. To study the influence of P-gp in tumor cell apoptosis, K562/A cells (P-gp+) and K562/S cells (P-gp−) were subjected to doxorubicin (Dox), serum withdrawal, or independent co-incubation with multiple P-gp inhibitors, including valspodar (PSC833), verapamil (Ver) and H108 to induce apoptosis. Apoptosis was simultaneously detected by apoptotic rate, cell cycle by flow cytometry and cysteine aspartic acid-specific protease 3 (caspase 3) activity by immunoassay. Cytotoxicity and apoptosis induced by PSC833 were evaluated through an MTT method and apoptosis rate, and cell cycle combined with caspase 3 activity, respectively. The results show that K562/A cells are more resistant to apoptosis and cell cycle arrest than K562/S cells after treatment with Dox or serum deprivation. The apoptosis of K562/A cells increased after co-incubation with each of the inhibitors of P-gp. P-gp inhibitors also enhanced cell cycle arrest in K562/A cell. PSC833 most strikingly decreased viability and led to apoptosis and S phase arrest of cell cycle in K562/A cells. Our study demonstrates that P-gp inhibits the apoptosis of tumor cells in addition to participating in the efflux of intracellular chemotherapy drugs. The results of the caspase 3 activity assay also suggest that the role of P-gp in apoptosis avoidance is caspase-related.

## 1. Introduction

Multidrug resistance (MDR) refers to a phenomenon in which tumor cells will develop resistance to a chemical agent during the course of treatment, and will no longer be responsive to that therapy, or multiple anticancer drugs that are functionally and structurally unrelated. This often leads to cancer relapse and eventually death of these patients [1]. Although scientists have done lots of research on MDR, the complicated mechanism of this phenomenon is not clear yet, and there is still not a clinically acceptable method or drug to effectively overcome MDR.

P-gp, a 170KD ATP-binding cassette (ABC) transporter, acts as a cell membrane pump extruding a wide variety of structurally unrelated xenobiotics to the extracellular space to reduce their accumulation in cells [2]. Most chemotherapy drugs are easily expelled from tumor cells since P-gp tends to be highly expressed on the cell surface of neoplastic tissue. P-gp has been proven to be one of the major drivers of MDR [3]. It has been proved that intracellular concentration of P-gp substrates, such as doxorubicin will increase at the existence of P-gp inhibitors, which can contribute to additional pharmacological effect [4,5].

Apoptosis is one of two cell death motifs, which is not only crucial for embryogenesis, development and homeostasis of the body, but also plays an important role in occurrence and development of tumors [6]. Lots of chemotherapy drugs exert therapeutic effects through inducing tumor cell apoptosis, and the progression of drug-induced apoptosis in neoplastic cells is a key measure that determines the effectiveness of chemotherapy drugs [7]. Therefore, the major goal of administering chemotherapy is to induce tumor cell apoptosis. Furthermore, recent research shows that the escape or antagonism of apoptosis by some mechanisms might lead tumor cells to become resistant to chemotherapy [8].

In addition to a reduction in tumor cell apoptosis by effluxing intracellular chemotherapy drugs, P-gp may also regulate cell proliferation, differentiation and death. For instance, aside from the elevated distribution in tumor cells and several organs [9], P-gp was also found in pluripotent stem cells, NK cells and early embryo-organs, where proliferation and apoptosis plays an important role [10,11]. P-gp can also transport some growth regulatory factors, such as IL-2 andIL-4, which play important roles in the growth and death of cells, especially in apoptosis [12]. P-gp-mediated MDR involves many signal transduction pathways and transcription factors which play important roles in apoptosis of cells [13]. All previous studies suggest that P-gp may affect death, especially apoptosis of cells through delicate regulative ways.

Additionally, it has also been reported that P-gp plays other roles in regulating death and apoptosis of tumor cells through methods other than efflux of intracellular chemotherapy drugs [14,15]. One such way is that P-gp possibly participates in the apoptosis mechanism. Moreover, researches implicated that P-gp regulates tumor cell apoptosis through the caspase dependent apoptosis pathway [16]. However, the evidence is rare and inconsistent [17,18].

In the present study, we systemically studied the influence of P-gp on apoptosis in different K562 cells with expression or in the absence of P-gp. The effects of P-gp inhibitors on apoptosis in these cells, and effects of P-gp inhibitor PSC833 on viability and inducing apoptosis in both cell lines were observed. The study of caspase 3 activity was also used to explore which apoptotic pathway P-gp played a role in.

## 2. Results and Discussion

### 2.1. Expression of P-gp in K562 Cells

**Figure 1:**
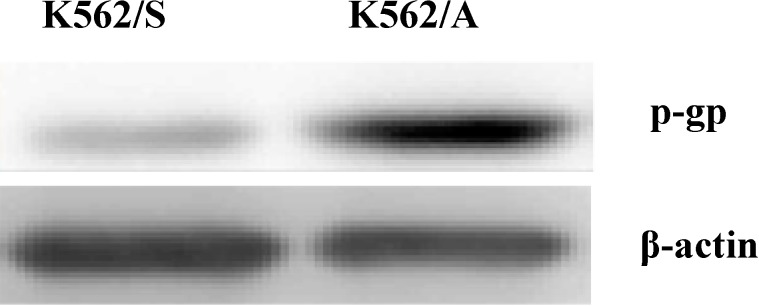
Expression of P-gp in K562 cells. Western blotting analysis of P-gp expression in K562/A cells and K562/S cells.

To analyze the P-gp expression in K562/A and K562/S cells in this study, western blotting was used (Figure 1). Results show that there was an obvious P-gp expression in the drug-resistant K562/A cells, whereas trace P-gp expression was found in K562/S cells. These results provide quality control for following comparison studies of tumor cell apoptosis in the P-gp positive and negative cell lines.

### 2.2. Apoptosis in K562 Cells Induced by Doxorubicin (Dox)

**Figure 2:**
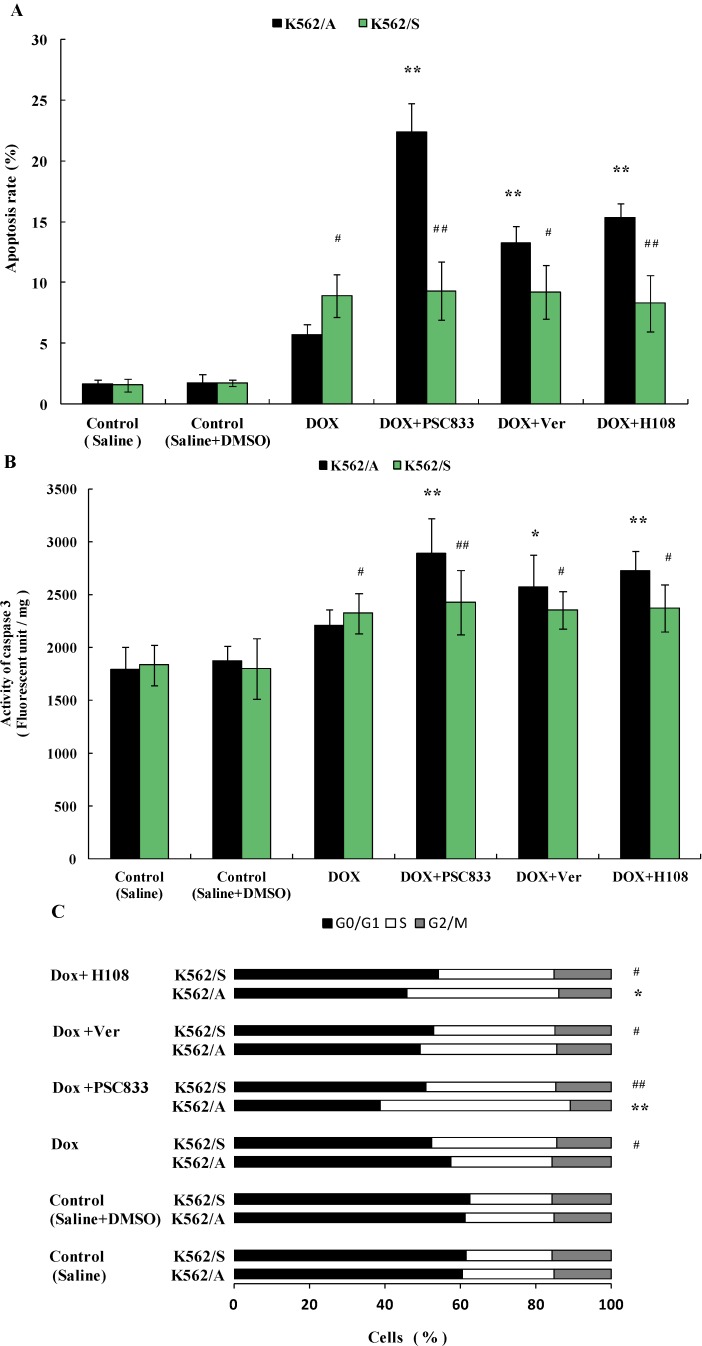
Apoptosis of K562/A and K562/S cells induced by Dox and effects of P-gp inhibitors. Both cell lines were incubated at the IC_30_ value of Dox (8.11 µM for K562/A and 0.016 µM for K562/S), some groups combined PSC833 (0.1 µM), Ver (5 µM) or H108 (5 µM), with Dox for 24 h. (**A**) Apoptotic rate of K562/A and K562/S cells determined by flow cytometry analysis. (**B**) Caspase 3 activity measured by immunoassay. (**C**) Cell cycle of K562/A and K562/S cells determined by flow cytometry analysis. Data are shown as mean ± SD. Student’s t-test (*n* = 6). * *p* < 0.05, ** *p* < 0.01 comparing with model (Saline + DMSO) group, # *p* < 0.05, ## *p* < 0.01 K562/S cells comparing with K562/A cells.

In an effort to explore the influence of P-gp on tumor cell apoptosis, apoptosis induced by Dox was observed in K562/A and K562/S cells, effects of different P-gp inhibitors on apoptosis in both cell lines were also studied. Apoptotic rate and cell cycle, combined with activity of caspase 3, was used to detect apoptosis. As DOX is a known substrate of P-gp, when adding it into P-gp expressing K562/A cells, DOX will be actively discharged out of cells, which will lead to the decrease of intracellular DOX concentration. As inducing apoptosis of K562/A and K562/S cells using the same working concentration of DOX may lead to different intracellular DOX concentrations, to minimize errors in the experiments, the same IC_30_ concentrations (30% inhibitory concentration, measured by MTT assay) of Dox for K562/A and K562/S were used in their respective cell lines according to preliminary experiments. The results show that after incubation with Dox for 24 h, the apoptosis rate of 5.72% in K562/A cells was significantly lower than 8.93% in K562/S cells (*p* < 0.05). Apoptotic rate of K562/A could be increased to 22.42%, 13.22%, or 15.37% (*p* < 0.01) when PSC833, Ver, or H108 was added, respectively, but these 3 P-gp inhibitors had no effect on the apoptotic rate in K562/S cells (Figure 2A). Meanwhile, when K562/A cells were incubated with Dox, caspase 3 activity increased 18.24%, which is significantly lower than that of K562/S (29.04%) (*p* < 0.05). When PSC833, Ver, or H108 were added, caspase 3 activity of K562/A cells further went up to 54.65% (*p* < 0.01), 37.60 (*p* < 0.05), or 45.79% (*p* < 0.01), respectively, whereas no significant changes in caspase 3 activity was observed when K562/S were treated with each of the P-gp inhibitors (Figure 2B). Following apoptotic trigger, both cells arrested in S phase of the cell cycle, which is accompanied by a decrease in the percentage of cells in G0/G1 phase. Compared with K562/S (33.1%), K562/A (26.8%) were more resistant to S phase arrest (*p* < 0.05). 

PSC833, Ver, or H108 further increased the percentage of cells in S phase to 50.3% (*p* < 0.01), 36.2%, or 40.2% (*p* < 0.05) in K562/A cells, respectively, while these P-gp inhibitors had no effect on cell cycle of K562/S cells (Figure 2C). These data suggest that P-gp leads tumor cells resistance to apoptosis.

### 2.3. Apoptosis of K562 Cells Induced during Serum Deprivation

**Figure 3:**
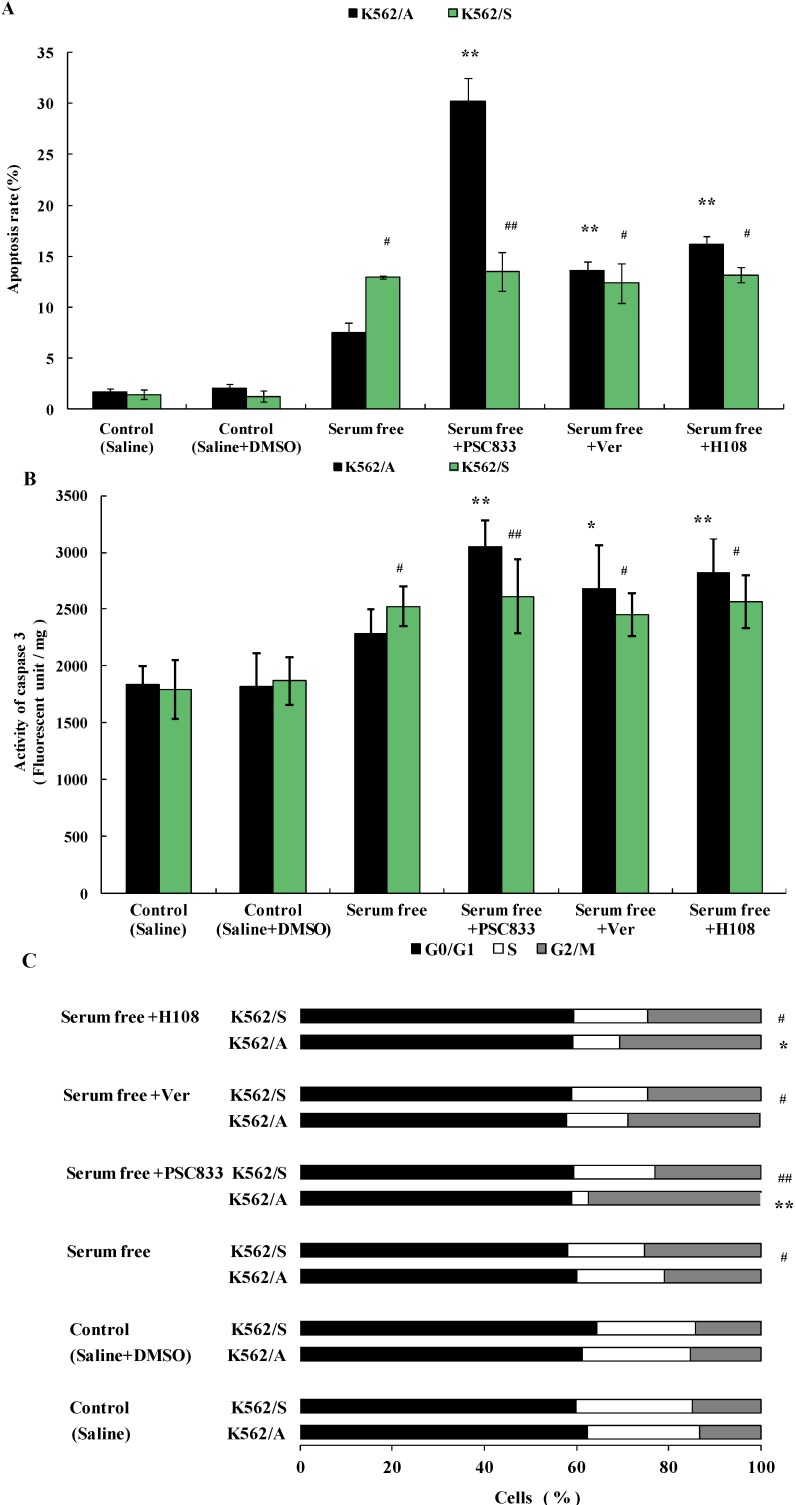
Apoptosis of K562/A and K562/S induced via serum deprivation and effects of P-gp inhibitors. Both cell lines were incubated with serum free culture medium, or in combination with PSC833 (0.1 µM), Ver (5 µM), H108 (5 µM), respectively for 48 h. (**A**) Apoptotic rate of K562/A and K562/S cells determined by flow cytometry analysis. (**B**) Caspase 3 activity measured by immunoassay. (**C**) Cell cycle of K562/A and K562/S cells determined by flow cytometry analysis. Data are shown as mean ± SD. Student’s t-test (*n* = 6). * *p* < 0.05, ** *p* < 0.01 comparing with model (saline + DMSO) group. # *p* < 0.05, ## *p* < 0.01 K562/S cells comparing with K562/A cells.

To further verify the relationship of P-gp and apoptosis in tumor cells, apoptosis of K562/A and K562/S cells were induced via serum deprivation. The results show the apoptotic rate of K562/S cells (12.92%) was significantly higher than that of K562/A cells (7.49%) (*p* < 0.05). PSC833 (30.23%), Ver (13.62%) or H108 (16.16%) significantly increased the apoptotic rate of K562/A cells (*p* < 0.01); meanwhile, PSC833, Ver, and H108 had no effect on the apoptotic rate of K562/S cells (Figure 3A). Similarly, caspase 3 activity of K562/A increased to 26.0%, lower than that of K562/S (35.26) (*p* < 0.05) after apoptosis was induced via serum deprivation. PSC833, Ver, or H108 further increased the caspase 3 activity of K562/A to 67.91% (*p* < 0.01), 47.47% (*p* < 0.05), or 55.16% (*p* < 0.01), respectively. No caspase 3 activity changes were observed in K562/S cells when apoptosis was co-incubated with each P-gp inhibitors (Figure 3B). Furthermore, the arrest of the cell cycle in G2/M with a concomitant decrease in the S phase was observed in the both cells after serum deprivation, K562/S group (25.2%) showed more significant G2/M arrest than k562/A cells (20.8%) (*p* < 0.05).

The proportion of k562/A cells in the G2/M phase further increased to 37.4% (*p* < 0.01), 28.8%, or 30.7% (*p* < 0.05), respectively when PSC833, Ver, or H108 was added, whereas no cell cycle changes were found when K562/S cells were co-cultured with P-gp inhibitors (Figure 3C). These results further demonstrate that the apoptosis can be hampered by P-gp, and this function is not based on its ability to efflux the drug.

### 2.4. Effects of PSC833 in Viability of K562 Cells

**Figure 4:**
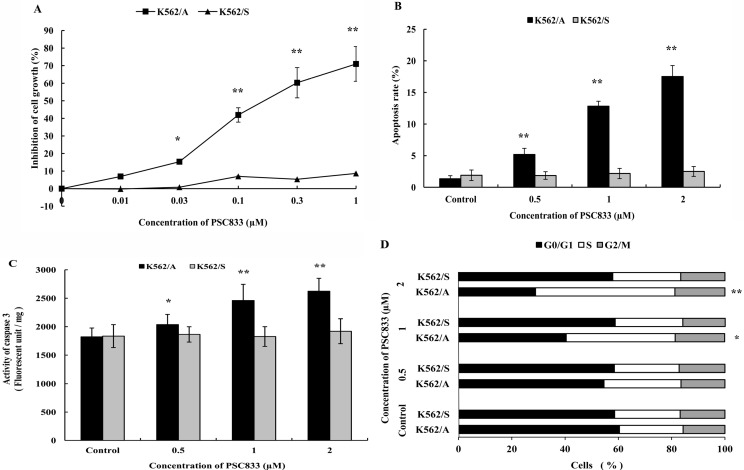
Effect of PSC833 on viability and induction of apoptosis in K562/A and K562/S. (**A**) Effect of PSC833 on viability of K562/A and K562/S. K562/A (■) and K562/S (▲) cells were treated with PSC833 (0.01–1 µM) for 72 h, then viability of cells was determined by MTT method; (**B**) Apoptotic rate of K562/A and K562/S induced by PSC833. Both cell lines were incubated with PSC833 (0.5, 1, 2 µM) for 48 h. Apoptotic rate was determined by flow cytometry analysis. (**C**) Caspase 3 activity induced by PSC833. Caspase 3 activity was measured by immunoassay. (**D**) Cell cycle of K562/A and K562/S cells determined by flow cytometry analysis. Data are shown as mean ± SD. Student’s t-test (*n* = 6). * *p* < 0.05, ** *p* < 0.01 comparing with control group.

To study tumor cell growth when P-gp is inhibited, effects of P-gp inhibitor PSC833 on viability of K562/A and K562/S cells were detected. After incubation with different concentrations of PSC833 for 72 h, viability of K562/A cells was significantly decreased in a dose dependent manner. However, no change in viability was observed in K562/S cell (Figure 4A). These results indicate that inhibition of P-gp can suppress tumor cell growth.

### 2.5. PSC833-Induced Apoptosis of K562 Cells

Based on the above viability test results, apoptosis of K562/A and K562/S cells were induced with the potent P-gp inhibitor PSC833 directly. These results show that after incubation with increasing concentrations of PSC833 for 48 h, obvious apoptosis was observed in K562/A cells in a concentration-dependent manner (*p* < 0.01). Whereas, no apoptosis was found in the K562/S cells (Figure 4B). Similarly, caspase 3 activity of K562/A was significantly increased to 11.85% (*p* < 0.05), 35.11% (*p* < 0.01) or 44.04% (*p* < 0.01) in K562/A also in a dose dependent manner, whereas no changes in caspase 3 activity were observed when K562/S cells were treated with any concentrations of PSC833 (Figure 4C). For the cell cycle analysis, Figure 4D shows that PSC833 treatment caused a time-dependent increase of the S-phase cell population to 28.9%, 40.9% (*p* < 0.05) or 52.3% (*p* < 0.01) in K562/A cells, with simultaneous decrease in G0/G1 phase cell population, suggesting that PSC833 induced cell cycle arrest at S-phase, whereas no obvious cell cycle changes were observed in K562/S after treatment of PSC833 (Figure 4D). This shows that inhibition of P-gp can directly lead to tumor cell apoptosis.

### 2.6. Discussions

While progress has been made in early detection, surgical resection, and targeted therapy, vastly improving survival rates for non-metastatic carcinoma, chemotherapy is still the main mode of treatment for invasive or metastatic cancer. Unfortunately, the MDR phenomenon is one of the main reasons that chemotherapy fails in the clinic, leading to poor survival for this group [19]. Therefore, the study of MDR mechanisms and therapies to overcome it is very important in cancer therapy and an important area of cancer research. Until now, the complete mechanism of MDR is unclear. Though it has been proven that the drug efflux pump P-gp is an important factor of MDR, many of the attempts at isolating one mechanism of MDR, such as inhibition of P-gp function alone, have encountered setbacks and faced enormous difficulties [20]. Scientists now see the resistance of tumor cells to apoptosis as another form of MDR [21] as MDR cells were found to resist apoptosis induced by many chemotherapeutics [22].

There has been abundant evidence suggesting that P-gp impacts apoptosis in tumor cells in recent years. For example, low concentrations of celecoxib were found to decrease P-gp expression, and conversely, increase the number of apoptotic cells [23]. Karwatsky *et al.* found that the P-gp inhibitor verapamil preferentially induced apoptosis in MDR cells with high P-gp expression compared to non-MDR cells [24], which indicates that inhibition of P-gp function is an effective way to induce apoptosis and overcome MDR. These studies suggested that there is a relationship between P-gp and apoptosis. It is generally believed that various mechanisms participate in MDR and several mechanisms operate in each tumor cell and interact to jointly promote the resistance to drugs as a network. Therefore, the disclosure of relationship between P-gp and apoptosis is important for studying MDR in tumor cells.

The Cysteinyl Aspartate Specific Proteinase (caspase) family is the apoptosis initiator and executor of mammalian cells [25]. Many apoptosis-inducing stimuli, such as chemotherapy drugs, serum starvation, TNF treatment, and others, induce apoptosis through the caspase-mediated pathway [26]. Caspase 3 is the most important effector caspase which is the core of the apoptotic process and is called as the “death protease”. Therefore, activation of caspase 3 is a key symbol of apoptosis [27]. To ensure the accuracy of apoptosis detection in this study, apoptosis rate and caspase 3 activity were analyzed simultaneously.

When apoptosis is induced by Dox, which has been proven to induce tumor cell apoptosis [28], P-gp positive tumor cells are more resistant to apoptosis than P-gp negative tumor cells, and different structural and functional classic P-gp inhibitors: PSC833 [29], verapamil [30] and a novel P-gp inhibitor H108 which was discovered in our laboratory [31] could enhance apoptosis of P-gp positive tumor cells. However, these P-gp inhibitors had no effect on the apoptotic rate for P-gp negative tumor cells (Figure 2). This indicates that expression of P-gp inhibits apoptosis in tumor cells. When apoptosis was induced with serum deprivation, another classic apoptosis-inducing method [32], or co-incubation with P-gp inhibitors, the similar results (Figure 3) further verified P-gp’s inhibitory effect on apoptosis in tumor cells and also proved that the influence of P-gp on apoptosis is not solely based on its efflux function. Moreover, our results that K562/A cells are more resistant to cell cycle arrest than K562/S cells and P-gp inhibitors increase these cell cycle arrest of K562/A cells in both experiments are consistent with the above results and further prove the action of P-gp in apoptosis. Since P-gp can inhibit apoptosis in tumor cells, apoptosis should occur when P-gp’s function is inhibited. As shown in Figure 4, a potent P-gp inhibitor PSC833 leads to cell death and directly induces apoptosis in P-gp positive tumor cells, which provides further evidence of the inhibitive effects of P-gp on apoptosis. As there are little studies in P-gp inhibitor induced apoptosis, especially PSC833 in apoptosis, the apoptotic pathway of PSC 833-induced apoptosis remains to be further studied.

It is of note that studies show that P-gp is involved in inhibition of caspase-mediated tumor cell apoptosis, whereas it has no influence on caspase-independent apoptosis [33,34]. Studies also found that P-gp can inhibit caspase 3 activation [35]. In our study, the caspase 3 activity of P-gp positive tumor cells was significantly enhanced when either P-gp inhibitors were co-incubated with apoptosis inducer or were used to induce apoptosis directly, whereas P-gp inhibitors had no effect on caspase activity in P-gp negative tumor cells (Figure 2, Figure 3 and Figure 4). These results are consistent with the above studies which suggest that P-gp related apoptosis is caspase-mediated. However whether P-gp affects caspase 3 directly (or through other upstream members of the caspase family, such as 1, 8, or 9) requires further study.

## 3. Experimental Section

### 3.1. Cell Culture

K562 cells are a human leukemia cell line which is commonly used in P-gp and MDR studies [36]. Drug-sensitive K562/S cells and the drug-resistant K562/A cells (Shanghai Cell Biology Institute, Shanghai, China) were cultured in RMPI1640 medium containing 15% calf serum. Dox (1 µg/mL) (Sigma-Aldrich, St.Louis, MO., USA) was added to the medium of K562/A cells to maintain resistance.

### 3.2. Western Blot Analysis of P-Glycoprotein Cells Culture

A 5 × 10^5^ cell pellet was lysed in 100 µL of Tris-HCL buffer (50 mM, pH7.4) containing PMSF (1 mM), leupeptin (1 µM), and pepstatin-A (1 µM). The cell suspension was vortexed twice and incubated on ice for 30 min. After centrifugation at 10,000× *g* for 10 min, the protein concentration in the supernatant was measured with a Bradford protein kit (Bio-Rad Co, Hercules, CA, USA). 50 µg of protein was loaded on a SDS-PAGE (T:C = 7:3.9) gel and run at 120 V for 2 h. Proteins were transferred onto a PVDF membrane with the electric transfer technique at 155 mA for 2.5 h. The membrane was washed twice for 10 min with PBS-T containing 0.2% Tween-20, followed by blocking with PBS-T buffer containing 5% non-fat milk for 1 h at room temperature. The incubation with 1:2,000 working dilution of the primary antibody in the blocking solution was performed overnight at 4 °C. The membrane was washed 3 × 5 min with PBS-T buffer and subsequently incubated with a 1:4000 dilution of the secondary antibody for 2 h at room temperature. After washing 3 × 5 min with PBS-T buffer, the membrane was exposed in the enzyme-catalyzed chemiluminescent (ECL) kit (NEN Life Science, Boston, MA, USA).

### 3.3. Apoptosis Induction

K562/S and K562/A cells (1 × 10^6^/mL) were seeded on 6-well plates. **A.** K562/A and K562/S cells were incubated with equivalent cytotoxic concentrations of doxorubicin hydrochloride (Dox, Sigma-Aldrich., 0.016 µM: IC30 for K562/S and 8.11 µM: IC30 for K562/A cells) or in combination with PSC833 (Sigma-Aldrich, 0.1 µM, ), verapamil hydrochloride (Ver, Sigma-Aldrich, 5 µM), H108 (China Pharmaceutical University, Nanjin, China, 5 µM). Cells were collected for analyses after 24 h of culture. **B.** Both cell lines were incubated with serum-free medium alone, or in combination with PSC833 (0.1 µM), Ver (5 µM), H108 (5 µM). Cells were collected for analysis after 48 h. **C.** K562/S and K562/A (1 × 10^6^/mL) were added to 6-well plates, PSC833 with final concentration of 0.5, 1 or 2 µM was added to both cell lines respectively, cells were collected for analyses after culturing for 48 h. In this and subsequent experiments, stock solutions were made by dissolving DOX and Ver in saline and by dissolving PSC833 and and H108 in dimethyl sulfoxide (DMSO). The final concentration of DMSO in the treated cells was less than 0.1% in all experiments. The single or mixed solvents were tested as control.

### 3.4. Apoptosis and Cell Cycle Analysed by Flow Cytometry

Cells were collected in the centrifuge at 1000× *g* for 5 min and then fixed with 70% cool ethanol at −20 °C overnight. The cells were centrifuged at 1000× *g* for 3 min and the deposit was washed three times with PBS buffer. Phosphoric acid-citric acid buffer (PCB, 0.5 mL) was used to resuspend cells at room temperature for 30 min to extract small molecular DNA. After the suspension was centrifuged at 1000× *g* for 3 min, the cells were stained for 30 min with propidium iodide (PI, Sigma-Aldrich.) staining solution. The cell suspension was filtered through nylon mesh to discard cell pellet. Apoptosis and cell cycle were analyzed using FacsCalibur Flow Cytometry (Becton Dicknson Co, Franklin Lakes, NJ, USA) with a 488 nm excitation wavelength and a 630 nm emission wavelength. Ten thousand cells were selected using CellQuest software (Becton Dicknson Co.) and then apoptotic cells were counted. The apoptosis peak was fitted and a distribution graph of DNA was drawn by using ModFit software (Verity Software House Co, Topsham, ME, USA), the G1 sub peak was considered the apoptosis peak.

### 3.5. Caspase 3 Activity Determined by Immunoassay

After removing medium by centrifugation, cells were lysed with cell lysis solution (0.5 mM NP-40, 20 mM Tris-HCL pH7.6, 120 mM NaCl, 2 mM benzamide, 10 mM NaF, 4 mM PMSF). Caspase 3 activity was examined by TruPoint Caspase-3 Assay Kit kit (Perkin Elmer Life Science Co, Waltham, MA., USA) as follows: Caspase-DTT-Reaction Buffer (10 μL) was pipetted into the reaction wells. TruPoint Caspase-3 Substrate (5 μL of 800 nmol/L) was added into the wells. The above cell lysates (5 μL) were added into the wells. The mixture is shaken slowly for 2 min and incubated for 60 min at 37 °C, protected from light. Fluorescence was measured at 340 nm for excitation and 615 nm for emission (1420 Victor Multi-label Immunoassay Analyzer. Perkin Elmer Life Science Co.). Amount of cells in each well was standardized with the protein quantity by Bradford assay Enzyme activity was expressed as fluorescence units per mg protein.

### 3.6. MTT Assay of K562 Cells after PSC833 Treatment

K562/A and K562/S cells (5 × 10^5^/mL) were collected and added to a 96-well plate. PSC833 (0.01, 0.03, 0.1, 0.3, 1 µM) was added to the cells, respectively. After 72 h, the viability of K562/A and K562/S cells was measured using the MTT method. In this test, the Inhibition Rate of Cell Growth (%) was defined as [A (drug) − A (model)]/[A (control) − A (model)] × 100%

### 3.7. Statistical Analysis

All data was presented as the mean ± standard deviation (mean ± SD). Student’s t-test was used for statistical analysis and statistical significance was defined as *p* < 0.05 or *p* < 0.01.

## 4. Conclusions

In this study, we systemically studied and demonstrated that P-gp is able to inhibit apoptosis of tumor cells besides effecting the efflux of intracellular chemotherapeutic drugs and our results are consistent with the previous studies which showed that P-gp-related apoptosis is caspase-dependent. These findings will be helpful in more in-depth studies of P-gp function, MDR mechanisms, and the role of apoptosis in MDR. It also suggests a novel and ideal approach for overcoming MDR.
